# Supplementary material for: SiONx Coating Regulates Mesenchymal Stem Cell Antioxidant Capacity via Nuclear Erythroid Factor 2 Activity under Toxic Oxidative Stress Conditions
Source: Antioxidants (Basel). 2024 Feb 1;13(2):189. doi: 10.3390/antiox13020189 (PMC10885901; doi:10.3390/antiox13020189)
Supplement: Supplementary file 1 [file antioxidants-13-00189-s001.zip › antioxidants-2778237-supplementary.pdf]

Supplemental Material

$SiON_x$ Coating Regulates Mesenchymal Stem Cell Antioxidant Capacity via NRF2 Activity Under Toxic Oxidative Stress Condition

Neelam Ahuja[1], Kamal Awad[1,2], Su Yang[3], He Dong[3], Pranesh Aswath[2], Simon Young[4], Marco Brotto[1], and Venu Varanasi[1*]

[1]Bone-Muscle Research Center, College of Nursing and Health Innovation, University of Texas at Arlington.

[2]Department of Material Science and Engineering, University of Texas at Arlington.

[3]Department of Chemistry and Biochemistry, University of Texas at Arlington.

[4]Department of Oral and Maxillofacial Surgery, University of Texas Health Science Center at Houston.

*Corresponding Author, (venu.varanasi@uta.edu)

Dr. Venu Varanasi, PhD

Associate Professor, Bone Muscle Research Center

College of Nursing and Health Innovation

University of Texas at Arlington

Address: 655 W. Mitchell St., Box 19410, Arlington, TX 76019

Email: Venu.varanasi@uta.edu

Phone: 817-272-1743

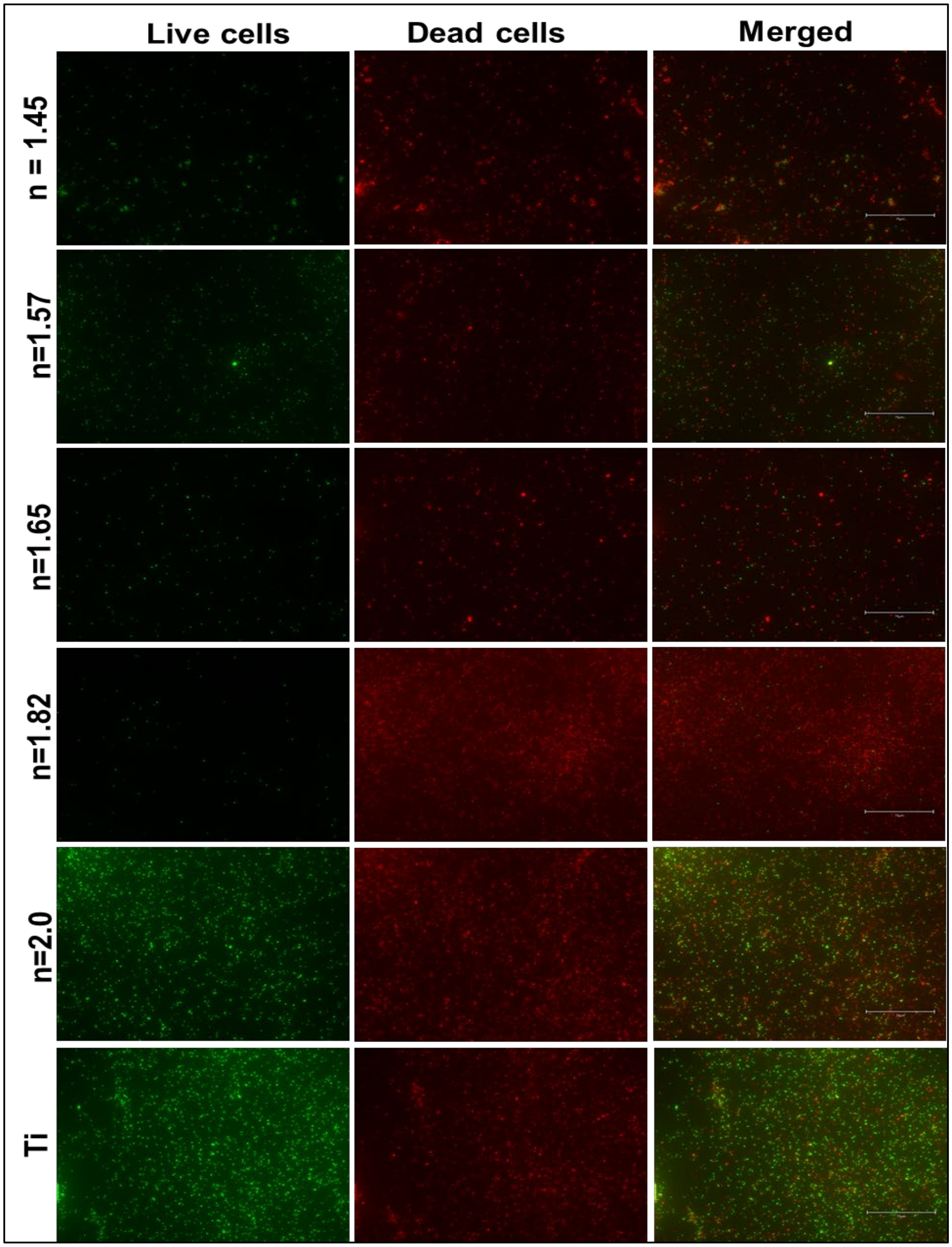

Supplemental Figure 1.1: Bacteriostatic effect of SiONx coatings verse Ti implants. Fluorescence images show the live (green) and dead bacteria (red) on the different SiONx surfaces compared to Ti implant after 12 hours.

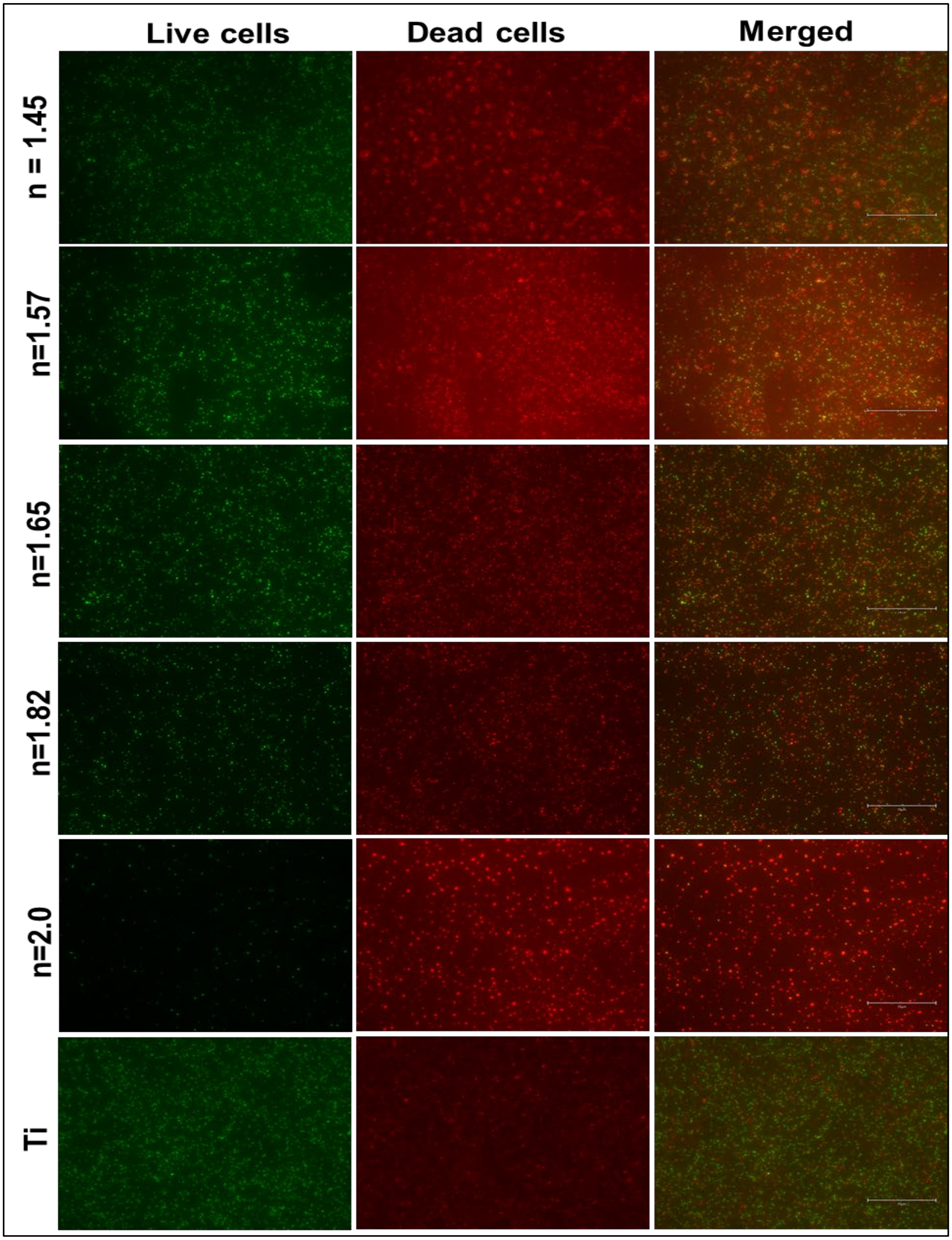

Supplemental Figure 1.2: Bacteriostatic effect of SiONx coatings verse Ti implants. Fluorescence images show the live (green) and dead bacteria (red) on the different SiONx surfaces compared to Ti implant after 24 hours.

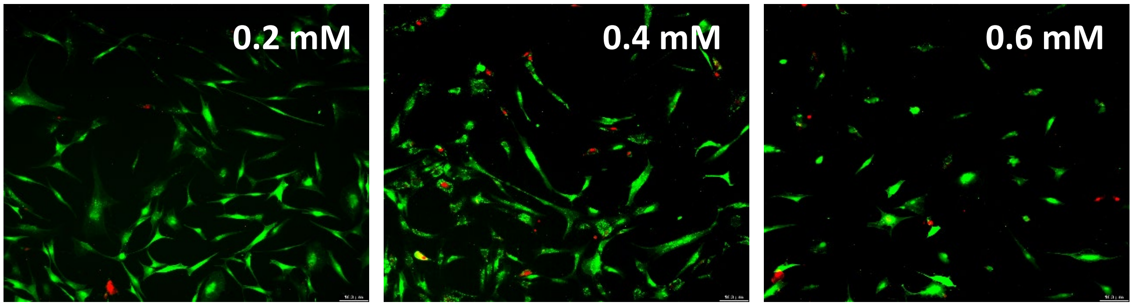

Supplemental Figure 2. Live/Dead assay performed after 4 days of an initial 24-hour insult using various concentrations of Hydrogen peroxide to study the sustained toxic effects of H_2O_2 on MSCs.

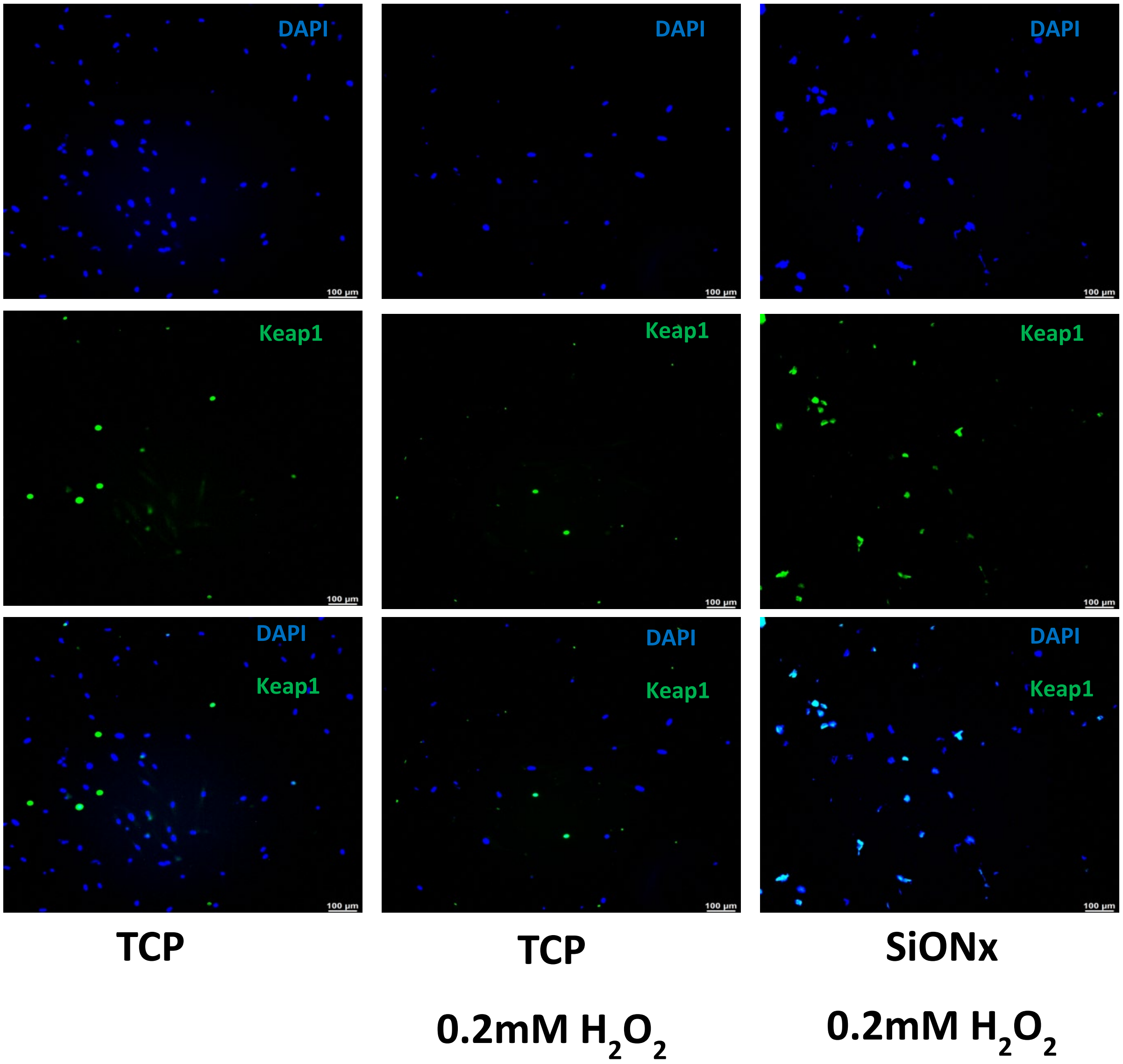

Supplemental Figure 3. IHC staining representing DAPI and Keap1 on MSCs after 7 days of osteogenic differentiation. There is no significant difference in the Keap1 activity on the SiONx surface when compared to the control.
